# Great debate: lipid-lowering therapies should be guided by vascular imaging rather than by circulating biomarkers

**DOI:** 10.1093/eurheartj/ehad275

**Published:** 2023-06-01

**Authors:** Lale Tokgozoglu, David A Morrow, Stephen J Nicholls

**Affiliations:** Department of Cardiology, Hacettepe University Faculty of Medicine, Ankara, Turkey; TIMI Study Group, Cardiovascular Division, Department of Medicine, Brigham and Women’s Hospital, Harvard Medical School, Boston, MA, USA; Victorian Heart Institute, Monash University, Melbourne, Australia

**Keywords:** Lipids, imaging, biomarkers, CT angiography, calcium score, Hs-CRP, Troponin, risk assesment

## Abstract

**Graphical Abstract:**
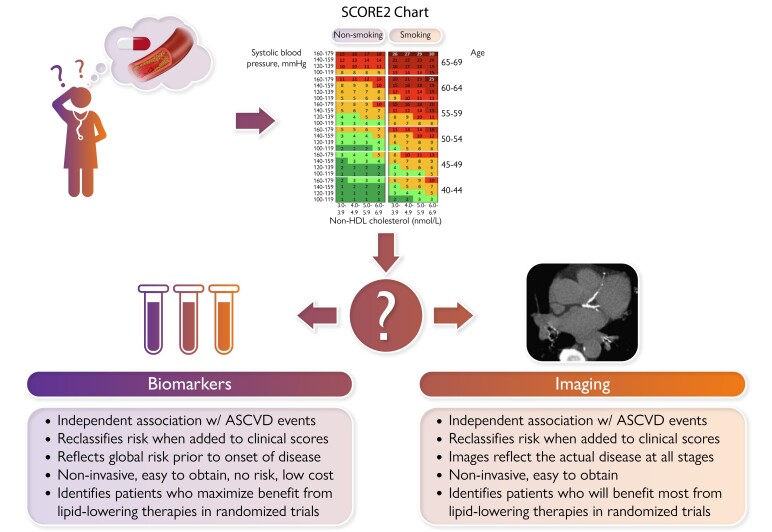
Advantages of tailoring lipid lowering therapy with biomarkers versus imaging.

Advantages of tailoring lipid lowering therapy with biomarkers versus imaging.

## Introduction

Dyslipidemia is an important modifiable risk factor for atherosclerotic cardiovascular disease (ASCVD) and lipid lowering an integral component of cardiovascular prevention. Apolipoprotein B containing lipoproteins—mainly low-density lipoprotein cholesterol (LDL-C)—have indubitably been shown to be causal for atherosclerosis, and interventions that lower LDL-C can change the trajectory of the disease to improve cardiovascular outcomes.^1^ With the ever-expanding armamentarium of lipid lowering therapies, it becomes important to decide to whom, when, and how to administer these therapies optimally. In the current guidelines, the decision to initiate and intensify lipid lowering therapy is guided by the untreated LDL-C levels of the individual as well as the total cardiovascular risk level.^2^ As the risk becomes higher, the actions are intensified in a graded manner. The risk of developing a cardiovascular (CV) event depends on the extent of atherosclerosis, which is the result of the complex interplay of genetics, lifestyle, and cumulative LDL-C levels over time. Current risk estimation systems consider major causal risk factors at a single time point to classify individuals into different risk categories. The most recent prevention guidelines in Europe use the contemporary and improved SCORE2 risk estimation system to determine the 10-year risk of CV events.^3^ However, this approach where risk estimation is based on group averages and applied to individual patients may not reflect genetic vulnerability or resilience, the cumulative exposure of risk factors over time, and interaction with other risk factors. While lifestyle should be recommended for all, there is a large group of patients in the moderate- or low-risk category where treatment decision needs more accurate assessment. Identifying novel risk markers may improve the selection of individuals for preventive strategies. Current risk estimation systems are limited to predicting 10-year risk therefore may underestimate the risk especially in women and younger individuals and overestimate risk in the elderly.^4^ A recent study applied the 2021 European guideline treatment criteria to a low-risk population and found that <1% of women met eligibility for class I recommendation to statins.^5^

In attempts to refine risk prediction further and tailor therapy in an optimal and cost-effective way, imaging and biomarkers have been utilized. The following debate will focus on whether biomarkers or imaging can help us answer the following questions better:

How can we better identify the seemingly low–moderate risk patient who will benefit from lipid lowering therapy and the high risk patient who needs treatment intensification?

Which tool will aid our decision to intensify or deescalate lipid lowering therapy?

Can imaging or a biomarker help us choose the ideal lipid lowering regimen in a given individual?

The 2021 European Prevention Guidelines base their treatment recommendations on plasma levels of LDL-C, apolipoprotein B, and non-HDL-C.^6^ Will adding any other biomarker help tailor therapy? Although not recommended in these guidelines, several other biomarkers have been utilized in an attempt to further define risk and personalize therapy. Recent evidence has shown that increased lipoprotein(a) [Lp(a)] leads to an incremental and continuous increase in absolute CV disease (CVD) risk.^7^ As Lp(a) levels increase, the LDL-C reduction needed to mitigate the increased risk of major CV events becomes higher, and elevated Lp(a) levels may justify more intensive LDL-C lowering therapy. Biomarkers of inflammation may also help guide therapy.^8^ High-sensitivity C-reactive protein (hs-CRP) >2 mg/L is considered a risk enhancer in the US and Canadian guidelines especially for intermediate risk patients.^9^ Newer lipid lowering therapies such as icosapent ethyl and bempedoic acid lower hs-CRP substantially, but whether this can be used to personalize therapy is not known. Other biomarkers, such as *N*-terminal pro-B-type natriuretic peptide and high-sensitivity cardiac troponin I, have also been shown to predict increased hazard of incident CVD and modestly improve discrimination and reclassification.^10^ Although biomarkers may help personalize therapy in selected patients, whether or when to use which biomarker in which patient is still debated.

On the other hand, imaging can give us the memory of lifetime exposure to risk factors. Non-invasive imaging can detect the presence, extent, and composition of the atherosclerotic plaque; all of which are determinants of CV events. Detection of coronary artery calcification with computed tomography (CT) improves both discrimination and reclassification for CV risk.^11^ The US multisociety guidelines on the management of blood cholesterol recommend to use coronary artery calcium (CAC) for guiding treatment decisions for primary prevention of ASCVD in individuals at borderline or intermediate risk.^12^ The European prevention guidelines consider CAC score as a risk modifier to reclassify CVD risk upwards or downwards in addition to conventional risk factors but exert caution about presence of noncalcified plaques that are not detected by CAC.^6^ Assessment of carotid or femoral plaque burden with ultrasound can also predict CV events and may be considered as a risk modifier in patients at intermediate risk when a CAC score is not feasible.^13^ Because of the cost, low-dose radiation, and need for specialized centers for some of these techniques, who will benefit most and at what stage of life from imaging need to be determined. The decision to utilize imaging or biomarkers should be personalized by carefully weighing the risk of testing—especially low dose radiation—against potential benefit of the intervention. Despite the supportive epidemiology, no randomized trial has yet directly tested the benefit of imaging-guided interventions on top of risk stratification using clinical characteristics and biomarkers. In the future, integrating a large number of patient-related variables over time with artificial intelligence including genetics, omics, biomarkers, imaging, and data from wearables can truly personalize lifetime risk prediction and management.^14^ Till then, we should utilize the tools we have to identify those who will benefit from lipid lowering meanwhile avoiding unnecessary overtreatment. The following debate will focus on whether imaging or biomarkers can better guide therapy today.

## Figures and Tables

**Figure 1:**
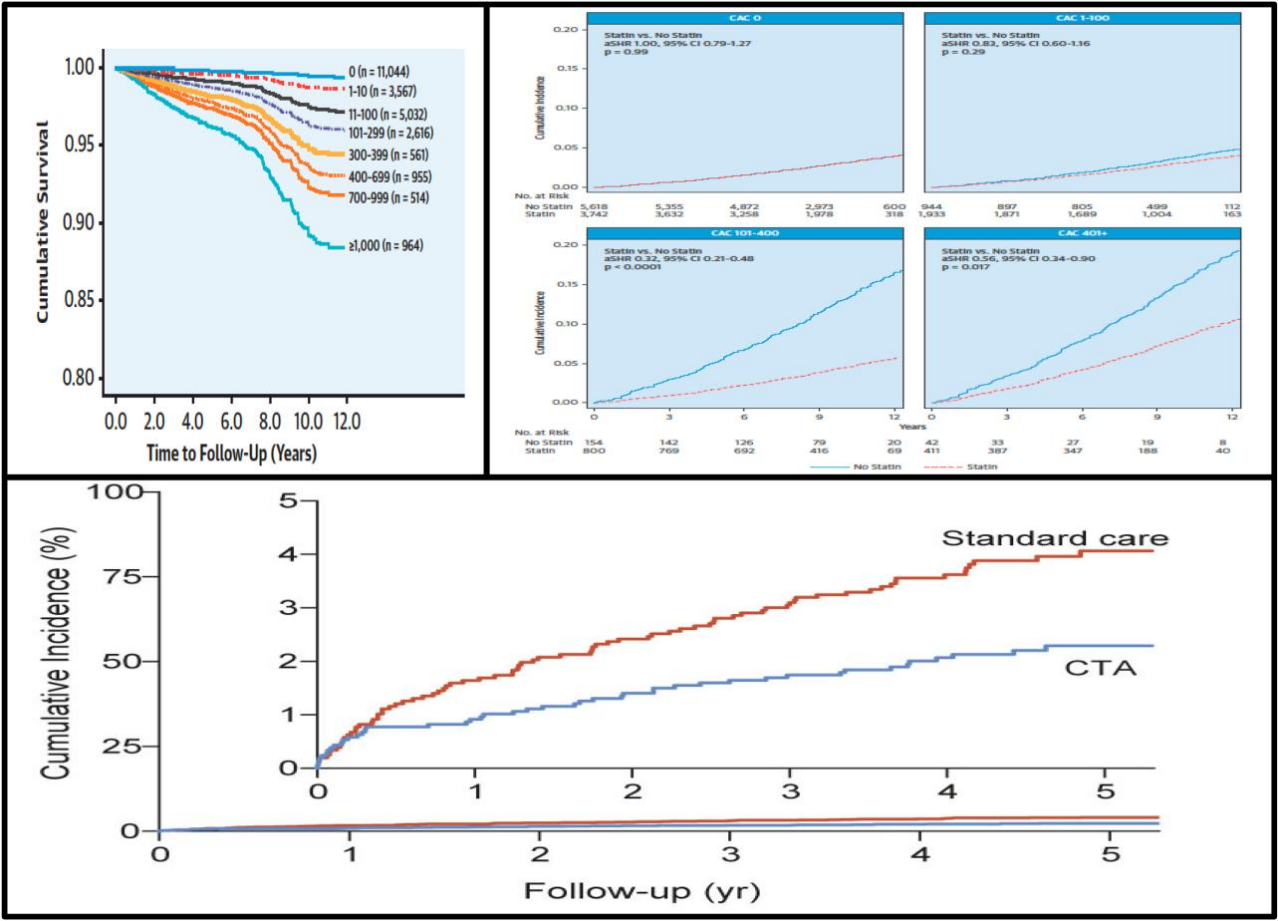
Computed tomography coronary imaging and cardiovascular risk. Relationship between increasing coronary calcium scores and cardiovascular risk (upper left panel). Impact of statin therapy on cardiovascular events in individuals with different calcium scores (upper right panel). Impact of triage to early computed tomography coronary angiography imaging on longer term cardiovascular events in patients with indeterminant chest pain (lower panel).

**Figure 2:**
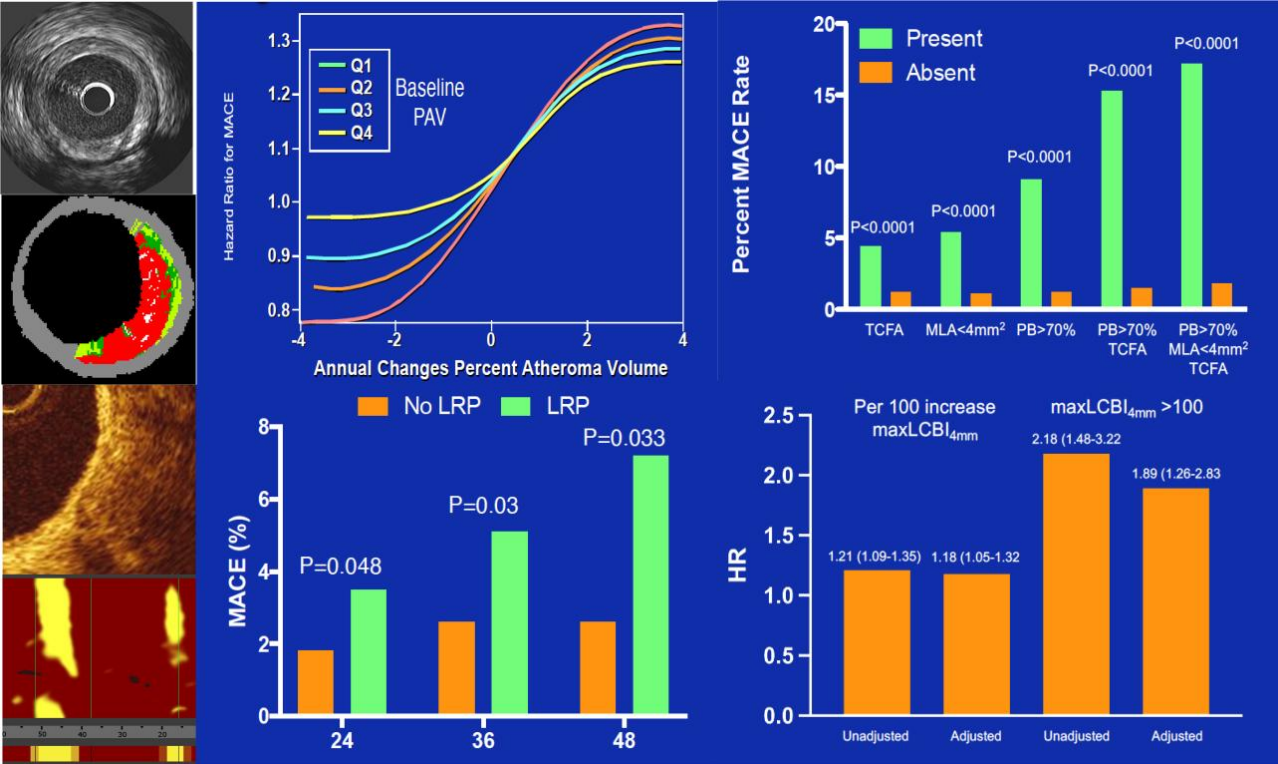
Invasive plaque imaging and cardiovascular risk. Examples of invasive coronary plaque imaging with intravascular ultrasound, virtual histology, optical coherence tomography, and near infrared spectroscopy (left panels). Relationship between plaque progression on intravascular ultrasound and cardiovascular risk (upper middle panel). Incremental cardiovascular risk prediction with a thin cap fibroatheroma on virtual histology (upper right panel). Relationship between finding of a lipid rich plaque on optical coherence tomography and cardiovascular events (lower middle panel). Relationship between plaque lipid content on near infrared spectroscopy and cardiovascular risk (lower right panel).

**Figure 1:**
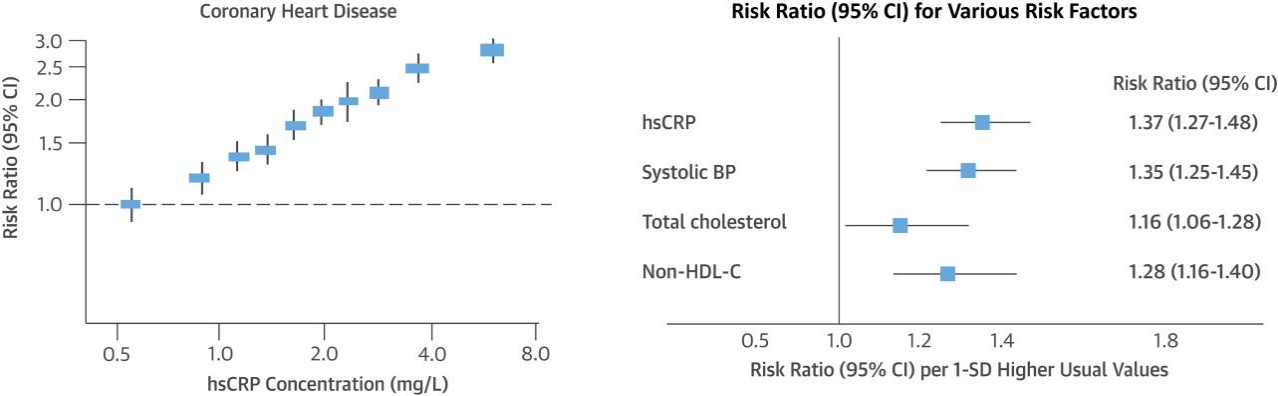
Relationship between high-sensitivity C-reactive protein concentration in healthy individuals and the future risk of coronary heart disease (left). The magnitude of risk associated with a 1-standard deviation (SD) change in high-sensitivity C-reactive protein is at least as great as that associated with a similar change in systolic blood pressure, total cholesterol, or non-high-density lipoprotein cholesterol (right). BP, blood pressure; CI, confidence interval; hsCRP, high-sensitivity C-reactive protein; HDL-C, high-density lipoprotein cholesterol. Adapted from Ridker (2016)^5^.

**Figure 2:**
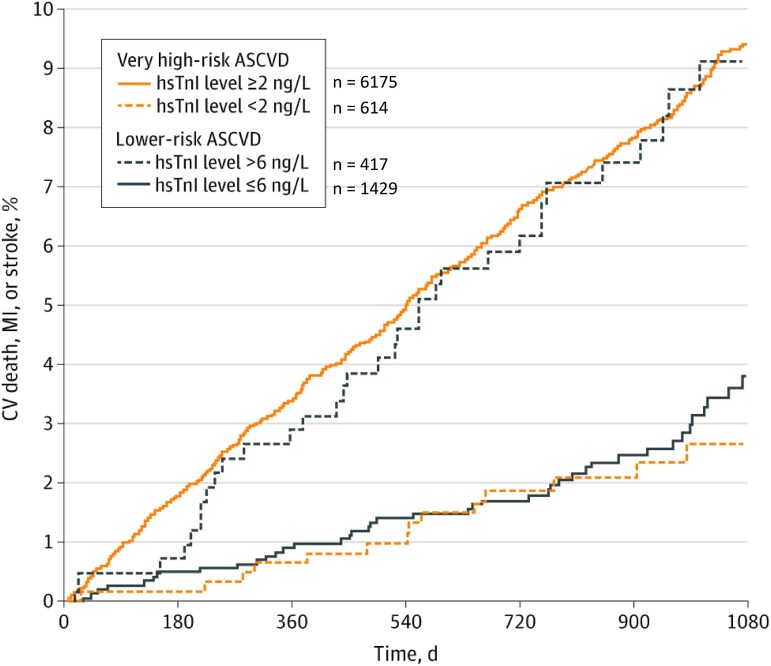
Reclassification of cardiovascular risk with the addition of high-sensitivity cardiac troponin in conjunction with the American Heart Association/American College of Cardiology management of blood cholesterol guidelines risk categories. ASCVD, atherosclerotic cardiovascular disease; CV, cardiovascular; MI, myocardial infarction. Adapted from Marston *et al.*^27^

**Figure 3:**
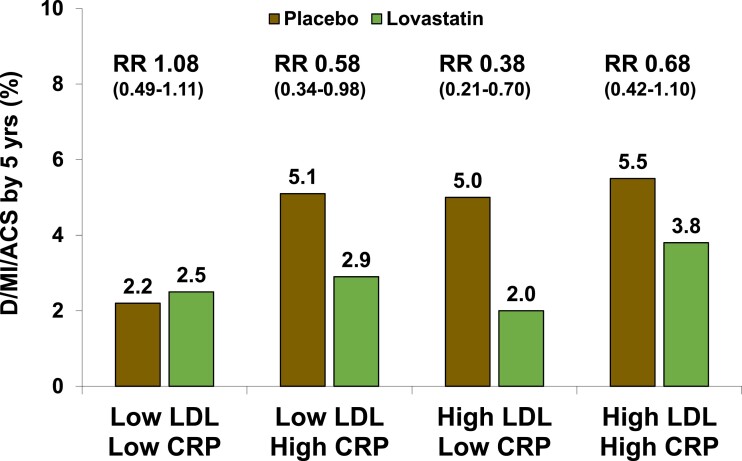
Effect of lovastatin vs. placebo on the rate of first acute coronary events, defined as myocardial infarction, unstable angina, or sudden death from cardiac causes, among 5742 men and women without known cardiovascular disease stratified by baseline C-reactive protein (high: >1.6 mg/L) and LDL-cholesterol concentration (high: >149 mg/dL). Data from Ridker *et al.*^29^

**Figure 4:**
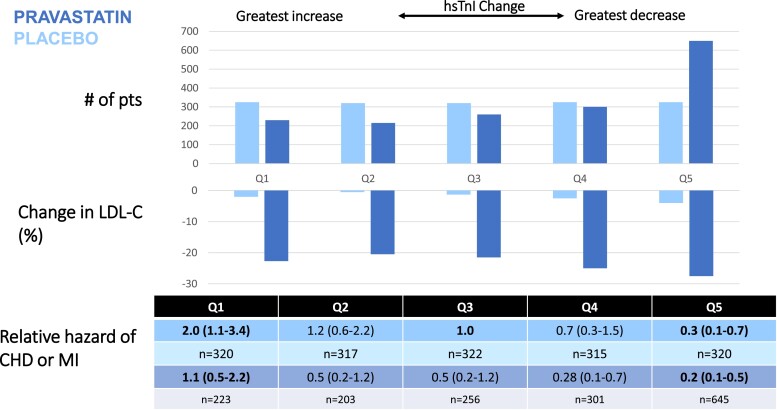
Change in high-sensitivity cardiac troponin and the risk of coronary heart disease death or myocardial infarction in primary prevention with pravastatin vs. placebo. Quartiles are for change in high-sensitivity troponin. Hazard ratios in the table are for the effect of pravastatin vs. placebo with the group treated with placebo who had no change in troponin (Q3) as the referent. Adapted from Ford *et al.*^18^

**Figure 5:**
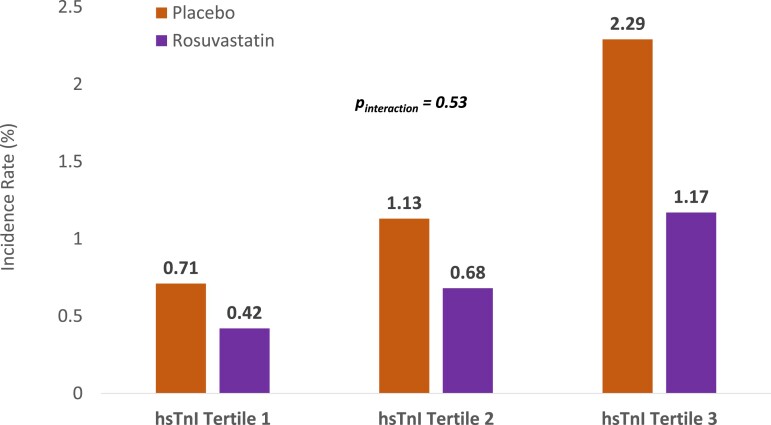
Incidence rate for the composite of nonfatal myocardial infarction, nonfatal stroke, hospitalization for unstable angina, arterial revascularization, or death resulting from cardiovascular causes for patients without prior known atherosclerosis treated with rosuvastatin vs. placebo stratified by baseline tertile of high-sensitivity troponin. Data are from Everett *et al.*^19^

**Figure 6:**
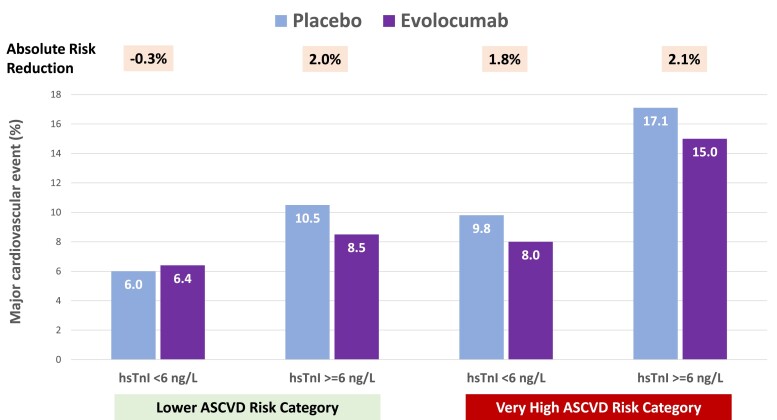
Effect of evolocumab vs. placebo on major vascular events (cardiovascular death, myocardial infarction, stroke, unstable angina or revascularization) in patients with known atherosclerosis stratified by baseline high-sensitivity troponin and American Heart Association/American College of Cardiology management of blood cholesterol guidelines risk categories. Data are from Marston *et al.*^36^

## Data Availability

No new data were generated or analysed in support of this research.

## Pro

https://orcid.org/0000-0002-9668-4368NichollsStephen J
Victorian Heart Institute, Monash University, Victorian Heart Hospital, 631 Blackburn Rd, Clayton, VIC 3168, Australia

Corresponding author. Email: stephen.nicholls@monash.edu

Treatment guidelines have placed lipid lowering as a cornerstone for strategies that reduce cardiovascular risk. While the use of intensive lipid lowering in the patient with clinically manifest cardiovascular disease is clear, the approach to identifying primary prevention patients with the greatest benefit of use of more intensive lipid lowering remains uncertain. In particular, the role of circulating biomarkers or vascular imaging to triage patients to more intensive lipid lowering remains an area of debate.

### Can simple circulating biomarkers alone triage patients to lipid lowering?

*Post hoc* analyses of lipid lowering trials suggest that a relationship between the degree of benefit of low-density lipoprotein cholesterol (LDL-C) lowering and baseline LDL-C level is not so simple. Investigation of studies involving comparisons of statin and placebo or higher and lower statin doses in the Cholesterol Treatment Trialists’ Collaboration revealed no difference in the relative risk reduction per mmol/L decrease in LDL-C, according to baseline LDL-C levels.^1^ In contrast, it was the overall risk of the patient that was most important—with the highest risk patients not only demonstrating a greater event rate, but also a greater absolute reduction in risk with lipid lowering.^2^ While additional biomarkers, such as high sensitivity measures of C-reactive protein^3^ or troponin,^4^ can predict cardiovascular risk in trials of lipid lowering, their upstream use does not necessarily identify the best therapeutic intervention to then apply.

### Importance of atherosclerotic plaque in cardiovascular risk

The concept that risk identifies patients who derive the greatest benefit from lipid lowering is important as it has major economic implications for health care systems. Ultimately, it is individuals deriving the greatest absolute risk reduction that constitute the smallest number needed to treat to prevent an event. The fact that acute ischemic events result from either rupture or erosion of an atherosclerotic plaque and the clear relationship between atherosclerotic disease burden and cardiovascular risk from multiple studies, including autopsy, invasive coronary angiography, intravascular imaging, and most recently computed tomography coronary angiography (CTCA),^5–11^ all support the argument that vascular imaging has the potential to play an important role in triaging patients to more intensive lipid lowering (*Figure 1*).

The potential benefit of intensive lipid lowering on risk attributable to atherosclerotic disease is supported by consistent findings from randomized clinical trials that have employed serial plaque imaging. Early studies using serial invasive coronary imaging have demonstrated a direct relationship between lowering levels of LDL-C and slowing progression of obstructive disease.^12,13^ Intravascular ultrasound^14^ permits accurate quantitation of plaque burden within the artery wall and has demonstrated a similar linear association between achieved LDL-C levels and the rate of progression of plaque volume, with evidence of increasing degrees of atheroma regression at LDL-C levels <70 mg/dL (1.8 mmol/L).^14–21^ The use of optical coherence tomography permits assessment of changes in plaque composition, with evidence that a greater degree of lipid lowering associates with greater thickening of the fibrous cap and a reduction in the size of the lipid pool.^20,21^ This suggests that more intensive lipid lowering has the potential to promote both regression and stabilization of coronary atherosclerosis. With advances in non-invasive imaging with CTCA, the benefits of lipid lowering on coronary atherosclerosis has been extended to asymptomatic cohorts.^22^ The demonstrated association between serial changes in coronary atherosclerosis and clinical outcomes further underscores the clinical importance of the findings of the studies.

### Calcium scoring to triage lipid lowering interventions

Non-invasive vascular imaging has the potential to identify subclinical atherosclerotic disease and triage individuals to therapies that are more likely to reduce their cardiovascular risk. Calculation of coronary calcium scores are well established to associate with the burden of cardiovascular risk factors, extent of coronary atherosclerosis, and subsequent risk of cardiovascular events.^23–26^ In particular, the ability of calcium scoring to reclassify cardiovascular risk in those individuals determined intermediate by conventional risk factor equations highlights its potential role in the clinic.^23,27^ This is further supported by observations that use of statins^28^ and aspirin^29^ are more likely to result in reductions in cardiovascular event rates in individuals with higher calcium scores—in fact, with little of evidence of benefit in individuals with calcium scores of zero (*Figure 2*). Health economic analyses further support the ability to use calcium scoring to triage individuals to the use of statin therapy. In a recent Australian analysis of individuals with a family history of heart disease, who do not currently meet the criteria for use of statins, application of different calcium score thresholds provided important insights into the cost effectiveness of guiding statin therapy. Initiation of statin therapy at any calcium score greater than zero would result in an increase in statin eligibility to 45%, with an incremental cost-effectiveness ratio of $53 028 per quality-adjusted life-year (QALY) gained. In contrast, applying a calcium score threshold of 100 for the use of statins would increase eligibility to 14% with greater cost effectiveness ($33 108 per QALY gained).^30^ The benefits are evident when applied to intermediate risk individuals, as opposed to population wide screening. The DANCAVAS study demonstrated that population-based computed tomography screening of men, aged 65–74 years, did not result in a reduction in all-cause mortality at 5 years.^31^

### Computed tomography coronary angiography to triage lipid lowering interventions

Advances in CTCA imaging permit the opportunity to characterize a range of features of atherosclerotic disease within the artery wall. These extend beyond simple measurements of luminal obstruction and plaque volume to include analysis of low attenuated plaque, fractional flow resistance as a physiological assessment of stenosis, and the degree of inflammatory activity within the perivascular adipose tissue. While each of these measures has been observed to associate with the risk of cardiovascular events, it is the ability to demonstrate that their use will alter clinical outcomes that has the greatest potential value. The SCOT-HEART study evaluated the impact of use of CTCA imaging or standard risk assessment in patients who presented with indeterminate chest pain. Those patients undergoing CTCA imaging, with the use of medical therapies guided by the presence of atherosclerotic disease, demonstrated a reduction of cardiovascular events over 5-year follow-up.^32^ The ability to use CTCA evidence of atherosclerotic plaque to guide the use of preventive therapies is undergoing further evaluation in the SCOT-HEART 2 (NCT03920176) study of 6000 asymptomatic, primary prevention patients with at least one risk factor. If a similar finding is observed in the original study, this will provide further evidence supporting the use of vascular imaging to guide the use of more intensive lipid lowering interventions.

### Clinical benefit of lipid lowering in patients with more extensive atherosclerotic disease

*Post hoc* analyses of lipid lowering studies such as the FOURIER study have consistently identified greater absolute risk reductions in patients at a higher risk of cardiovascular events. Subsequent analyses of patients with either recurrent ischemic events, multivessel coronary artery disease, or clinical atherosclerotic disease involving multiple vascular territories demonstrated not only higher cardiovascular event rates but also a greater absolute risk reduction with evolocumab, compared with patients without these clinical settings.^33,34^ Similarly, analyses of the studies establishing the benefits of coronary artery bypass surgery compared with medical therapy found this relationship to be strongest in patients with more extensive disease on baseline angiography.^11^ A common underlying factor in each of these settings involves the presence of more extensive and diffuse atherosclerotic disease, which complements observations from serial imaging of greater plaque regression with lipid lowering in patients with the greatest amount of atheroma at baseline.^35^ These observations provide further support for the concept that those individuals with more extensive disease derive a greater clinical benefit with intensive lipid lowering and for the potential clinical benefit that can follow the use of vascular imaging.

### Studies of the impact of vascular imaging

Observational studies from the Multi-Ethnic Study of Atherosclerosis demonstrate that the presence of an elevated coronary calcium score is associated with either greater initiation of preventive therapies (aspirin, lipid, or blood pressure lowering therapies) or greater likelihood that they will be continued if currently used.^36^ Furthermore, studies of individuals aged 40–60 years with at least one cardiovascular risk factor demonstrated that presentation of risk factor advice in combination with being shown their carotid ultrasound imaging results in a greater reduction in risk factor scores compared with not being shown imaging results.^37,38^

### Limitations

The potential benefits of lipid lowering interventions, guided by the findings of vascular imaging, continue to accumulate. This is reflected in the inclusion of vascular imaging in prevention guidelines. However, a number of caveats should be noted. A calcium score of zero does not eliminate the risk of cardiovascular disease, rather it is important in its ability to reclassify an individual’s cardiovascular risk to a lower level. It should be noted that this is less useful in younger individuals and in specific clinical settings, such as genetic dyslipidemia, where greater evidence is required to understand its true utility. Any form of computed tomography imaging involves radiation exposure, and in the setting of CTCA, not all patients are satisfactory candidates (renal failure, atrial fibrillation, obesity, and contrast allergy) for its use. While some evidence for cost effectiveness exists, this requires further investigation in different health care systems. As much of the evidence was originally derived from observational studies, the ability to prospectively demonstrate that application of imaging changes management and ultimately clinical outcomes in randomized clinical trials provides a much stronger case for its use.

### Summary

Increasing evidence suggests that more intensive lipid lowering leads to a greater clinical benefit in those individuals with more extensive atherosclerotic disease. With increasing ease of use and information derived from vascular imaging, the results of clinical studies and integration with prevention guidelines, suggest that there is a role for its use to guide the use of lipid lowering interventions. Ultimately, if it is an atherosclerotic plaque that causes an ischemic event and now we have the ability to identify those plaques with imaging, there is an ideal opportunity to treat them.

### Data availability

No new data were generated or analysed in support of this research.

## Contra

https://orcid.org/0000-0002-9589-5382MorrowDavid A
TIMI Study Group, Cardiovascular Division, Department of Medicine, Brigham and Women's Hospital, Harvard Medical School 75 Francis St, Boston, MA 02115, USA

Corresponding author. Email: dmorrow@bwh.harvard.edu

Current ESC/EAS guidelines for the management of dyslipidaemias establish estimation of total cardiovascular risk (for atherosclerotic cardiovascular disease [ASCVD]) as the foundation for decision-making regarding lipid-lowering therapies.^1^ Such risk stratification integrates the combined effect of individual risk factors and considers the global risk for the patient rather than lesion- or anatomy-specific risk. The ASCVD is a systemic disease that is not limited to any specific arterial bed. As such, tools for risk stratification that *reflect systemic processes* contributing to ASCVD are inherently desirable to inform a global estimation of risk for the individual patient. Such risk indicators may ascertain either processes that contribute to or are the result of the development and progression of ASCVD. They should be easy to measure and obtained at relatively low cost. Moreover, a central tenet in primary prevention is to mitigate risk before the development of disease.^2^ In contrast, tools that focus on specific anatomic manifestations of ASCVD may provide enhanced disease specificity but may miss important risk indicators in some patients and only identify atherosclerosis once it is present. Moreover, when associated with greater cost and the potential for harm from ionizing radiation, imaging approaches focused on anatomic manifestation of ASCVD are better used as second-line testing in patients with an estimated moderate total risk based on historical risk factors *and* cardiovascular biomarkers. Ultimately, to justify the use of more costly imaging approaches, data from randomized trials are needed, but, as yet, lacking, to demonstrate incremental value of vascular imaging to target therapeutic strategies for lipid lowering.

Current ESC/EAS guidelines for the management of dyslipidaemias establish estimation of total cardiovascular risk (for atherosclerotic cardiovascular disease [ASCVD]) as the foundation for decision-making regarding lipid-lowering therapies.^1^ Such risk stratification integrates the combined effect of individual risk factors and considers the global risk for the patient rather than lesion- or anatomy-specific risk. The ASCVD is a systemic disease that is not limited to any specific arterial bed. As such, tools for risk stratification that *reflect systemic processes* contributing to ASCVD are inherently desirable to inform a global estimation of risk for the individual patient. Such risk indicators may ascertain either processes that contribute to or are the result of the development and progression of ASCVD. They should be easy to measure and obtained at relatively low cost. Moreover, a central tenet in primary prevention is to mitigate risk before the development of disease.^2^ In contrast, tools that focus on specific anatomic manifestations of ASCVD may provide enhanced disease specificity but may miss important risk indicators in some patients and only identify atherosclerosis once it is present. Moreover, when associated with greater cost and the potential for harm from ionizing radiation, imaging approaches focused on anatomic manifestation of ASCVD are better used as second-line testing in patients with an estimated moderate total risk based on historical risk factors *and* cardiovascular biomarkers. Ultimately, to justify the use of more costly imaging approaches, data from randomized trials are needed, but, as yet, lacking, to demonstrate incremental value of vascular imaging to target therapeutic strategies for lipid lowering.

### Cardiovascular biomarkers for assessment of total cardiovascular risk

In addition to specific circulating lipids, including low-density lipoprotein cholesterol (LDL-C), apolipoprotein B, and lipoprotein(a), that are risk factors for ASCVD, other circulating cardiovascular biomarkers also show graded independent associations with the risk of first and future ASCVD events. Biomarkers of inflammation, hemodynamic stress, and myocardial injury each can reflect underlying systemic and, in the case of cardiac troponin, cardiac-specific processes that are either the cause or consequence of ASCVD.

Inflammation has been established to play a role in all stages of the initiation, development, and progression of ASCVD.^3^ Buffon *et al.*^4^ demonstrated two decades ago that even in unstable ASCVD, vulnerability in the coronary bed is a diffuse process. Inflammatory biomarkers, including the prototypical C-reactive protein (CRP), can reflect this global risk. Despite a lack of specificity for the cause of inflammation, data from multiple epidemiologic studies have established an independent association between elevated serum or plasma concentrations of CRP and the prevalence of underlying atherosclerosis, the risk of recurrent ASCVD events, and the incidence of first events among individuals at risk for ASCVD (*Figure 1*).^5^ When applied in the general population, the incremental predictive information from CRP is quantitatively small. However, when assessed in patients with intermediate risk, CRP offers meaningful additional information for risk stratification. For example, in the Framingham Offspring Study, among 3006 patients without ASCVD followed for an average of 12 years, after adjusting for traditional risk factors, patients with CRP >3 mg/L vs. CRP <1 mg/L had a nearly two-fold higher risk of myocardial infarction or coronary heart disease-related death [hazard ratio (HR) 1.88, 95% confidence interval (CI) 1.18–3.00] and a more than 1.5-fold higher risk of ASCVD events (coronary heart disease, stroke, transient ischemic attack, or claudication; HR 1.58, 95% CI 1.16–2.15).^6^ In a meta-analysis from the Emerging Risk Factors Collaboration incorporating 246 669 persons without prior ASCVD from 52 prospective studies, the addition of CRP concentration to traditional risk factors improved the risk assessment with a small but significant net reclassification improvement.^7^ In contrast, in the Women’s Health Study, CRP reclassified 20% of women categorized initially as intermediate risk using standard risk models.^8^ As such, measurement of high-sensitivity CRP for individuals with intermediate cardiovascular risk as determined using traditional risk factors is a practical tool that can be applied using available risk calculators^9,10^ and is included in the American College of Cardiology/American Heart Association cholesterol guidelines as an additional risk indicator that may be considered to inform treatment decisions.^11,12^

More recently, the epidemiology of biomarkers that may reflect consequences of ASCVD has been studied for evaluation of ASCVD risk in stable patients and those at risk for ASCVD. In particular, high-sensitivity cardiac troponin (hs-cTn) assays reveal measurable cTn in the blood of most individuals with values within the normal range demonstrating prognostic importance for relevant outcomes including coronary heart disease, stroke, and fatal cardiovascular disease^13–16^ One potential explanation is that hs-cTn reflects subtle abnormalities in individuals with otherwise unrecognized cardiovascular comorbidities. This concept is supported by evidence that more extensive screening that eliminates comorbidities detected with other biomarkers and imaging progressively lowers the upper value of the normal range.^17^ When applied among patients at risk for ASCVD in the West of Scotland Coronary Prevention Study, individuals with hs-cTn in the highest quartile were at more than two-fold higher risk of a first myocardial infarction or coronary heart disease-related death over 5 years (HR 2.3, 95% CI 1.4–3.7).^18^ Similarly, among 12 956 primary prevention candidates, hs-cTn in the highest tertile was associated with a doubling of the risk of a first major cardiovascular event (adjusted HR 2.19; 95% CI 1.56–3.06).^19^ Moreover, hs-cTn also demonstrates strong prognostic performance in patients with stable ASCVD.^20–26^ When applied in concert with the risk classification from the 2018 AHA/ACC cholesterol management guidelines, hs-cTn values delivered information that was complementary to the 13 clinical risk factors in the AHA/ACC guideline risk algorithm and reclassified 20%–25% of not very high-risk patients into a group whose risk profile is similar to the ACC/AHA very high-risk group and would be considered for additional lipid-lowering therapy in the ACC/AHA guidelines (*Figure 2*).^27^

Each of these biomarkers is inexpensive to measure, particularly in comparison to vascular imaging, and can be obtained during a routine patient visit without the need for any special procedures or exposure to radiation. When used in combination with clinical instruments for risk stratification, such cardiovascular biomarkers are most useful for incremental risk classification in those stratified to moderate risk who do not otherwise qualify for lipid-lowering therapy. Use of more than one biomarker in combination (e.g. CRP, hs-cTn, and a natriuretic peptide) may further enhance discrimination and net reclassification (e.g. ∼10%).^28^ They may be used as a ‘gate-keeper’ to more costly testing when risk remains uncertain after integration with clinical scores recommended by professional society guidelines.

### Cardiovascular biomarkers and lipid-lowering therapies

In addition to the established prognostic information for ASCVD offered by selected cardiovascular biomarkers, their demonstrated interplay with lipid-lowering strategies is even more important to their role in clinical decision-making. Based on nested studies from randomized trials, both CRP and hs-cTn identify higher risk patients who have the most to gain from lipid-lowering therapy and show reductions in concentration in conjunction with therapeutic efficacy.

First considering CRP, in the AFCAPS/TexCAPS trial of primary prevention with lovastatin, CRP identified 25% of patients with an LDL-C concentration below the median but CRP above the median who experienced a 42% relative reduction in acute coronary events with statin therapy vs. placebo (number needed to treat 48) comparing favorably to those with LDL above the median with low CRP (number needed to treat 33; *Figure 3*).^29^ Moreover, in this randomized trial, compared with placebo, lovastatin reduced CRP by almost 15%. Subsequently, the JUPITER trial prospectively tested the hypothesis that primary prevention patients with below average LDL-C (<3.4 mmol/L) and elevated hs-CRP (≥2 mg/L) would benefit from stain therapy vs. placebo. JUPITER enrolled 17 802 patients who were randomly assigned to treatment with either rosuvastatin 20 mg daily or placebo and demonstrated a 44% relative reduction in major cardiovascular events with statin therapy (HR 0.56; 95% CI 0.46-0.69, *P*<0.00001).^30^ This notion of using CRP to identify candidates for specific lipid-lowering strategies is also supported by trials among patients with established ASCVD. Among 4162 patients in the PROVE IT-TIMI 22 trial who were randomized to atorvastatin 80 mg/day or pravastatin 40 mg/day, the level of CRP after 30 days of therapy was linearly related to the risk of recurrent myocardial infarction or coronary death despite minimal correlation between the achieved levels of CRP and LDL-C.^31^ The concept of targeting CRP as a modifiable indicator of global risk for ASCVD primary and secondary events is further supported by the observation of superior outcomes in multiple randomized trials among patients achieving ‘dual’ goals of lowering both LDL-C and CRP with statin therapy.^32–34^

Analogously for hs-cTn, several lines of evidence are emerging that hs-cTn may be useful for decision-making regarding preventive therapies. In the related domain of anti-hypertensive therapy for primary prevention, hsTn substantially reclassified risk beyond blood pressure alone and better identified individuals who should receive antihypertensive therapies using risk categories identified by the 2017 ACC/AHA blood pressure guidelines.^35^ Taking a similar approach, hsTn may be useful for identifying patients who benefit most from LDL-C lowering therapies. For example, in the West of Scotland Coronary Prevention Study, the greatest absolute benefit with pravastatin vs. placebo was apparent among individuals with hs-cTn values that declined significantly compared with those whose hs-cTn did not (*Figure 4*). In the JUPITER trial, while there was no heterogeneity in the relative risk reduction with rosuvastatin vs. placebo, the absolute risk reduction went from 0.30 to 1.12 per 100 person-years from the first to third tertile of hs-cTn (*Figure 5*).^19^ In addition, among patients with stable ASCVD, measurement of hsTn might easily and affordably identify patients who are at low, intermediate, and high risk of recurrent ASCVD events. The hs-cTn concentrations well below the level used to diagnose acute myocardial infarction can stratify patients with stable ischemic heart disease into low risk (<1%/year), intermediate risk (1%–3%/year), and high risk (>3%/year) cohorts. Compared with the intermediate (average) risk patient, hsTn identifies both individuals with higher hs-cTn who are at two-fold higher risk of ASCVD events, as well as individuals with hs-cTn that is non-detectable and are at very low risk of future events.^27^ By reclassifying patients who appear to be a lower risk using clinical risk criteria alone, an approach using hs-cTn identifies 20%–25% of lower risk ASCVD patients who actually carry an annualized ASCVD event risk similar to the ACC/AHA very high-risk group and might be considered in the ACC/AHA guidelines for ezetimibe or PCSK9 inhibition in the same way as very high-risk patients.^27^ Moreover, in an analysis of >22 000 ASCVD patients, hsTn-based selection of patients who would not otherwise be a candidate for a PCSK9 monoclonal antibody reclassified patients who derived significant benefit from evolocumab vs. placebo with a relative risk reduction of 20% and an absolute risk reduction of 2.0% (*Figure 6*).^36^ Such analyses suggest that incorporating hs-cTn as an inexpensive and widely available biomarker into ASCVD risk assessment could both improve risk stratification, and more importantly, ensure patients are offered risk-appropriate medical therapies.

### Limitations of imaging

In addition to the obvious limitations of cost and radiation exposure for computed tomography (CT)-based imaging, it is important to note that coronary artery calcium (CAC) score is less sensitive to ASCVD in younger patients (e.g. <60 years old),^37^ including among very high risk individuals due to severe familial hypercholesterolaemia.^1^ At the same time, in older adults, the high prevalence of coronary calcification reduces specificity, and coronary CT is subject to reduced accuracy due to overestimation degree of stenosis with a higher prevalence of vascular calcification.^38^ Moreover, CAC score is increased following statin treatment; therefore, the CAC scores of statin-treated patients should be interpreted with caution. Finally, the use of imaging techniques, particularly with CT, is not justified in low-risk individuals due to poor prognostic yield, and the counter-balancing costs and radiation hazard.^1^

### Summary

Although reclassification and discrimination appear superior with vascular imaging compared with biomarkers in some studies, the low cost, low risk, and ease of ascertainment favors the first line adjunctive use of circulating biomarkers, which also have established independent associations with outcomes and demonstrated ability to identify patients with greater absolute magnitude of benefit in randomized trials of lipid-lowering therapies. Biomarkers are able to open a window into total risk, reflecting systemic processes with the possibility to prevent atherosclerosis before you can see it.

### Data availability

No new data were generated or analysed in support of this manuscript, all data are referenced.
